# Increasing HIV Incidence among Men Who Have Sex with Men in Jiangsu Province, China: Results from Five Consecutive Surveys, 2011–2015

**DOI:** 10.3390/ijerph13080795

**Published:** 2016-08-06

**Authors:** Haiyang Hu, Xiaoyan Liu, Zhi Zhang, Xiaoqin Xu, Lingen Shi, Gengfeng Fu, Xiping Huan, Ying Zhou

**Affiliations:** Jiangsu Provincial Center for Disease Control and Prevention, Nanjing 210009, China; huhaiyang@jscdc.cn (H.H.); liuxy@jscdc.cn (X.L.); zhangzhi@jscdc.cn (Z.Z.); xuxiaoqin@jscdc.cn (X.X.); yylen2006@163.com (L.S.); fugf@jscdc.cn (G.F.); huanxp@vip.sina.com (X.H.)

**Keywords:** HIV incidence, IgG-capture BED-EIA, men who have sex with men, China

## Abstract

Epidemics of HIV among men who have sex with men (MSM) are major public health concerns in most parts of China. This study examined the trends in HIV incidence and associated factors among MSM in Jiangsu Province. Five consecutive cross-sectional surveys were conducted among MSM from 2011 to 2015 in eight cities throughout Jiangsu Province. Participants were recruited from MSM venues or via the internet. Demographic and behavioral data were collected through HIV bio-behavioral surveys. Blood specimens were collected to test for HIV and syphilis. HIV incidence was estimated by the IgG-capture BED-EIA (BED) method and a chi-square trend test was used to compare differences over the years. Multivariate logistic regression analysis was used to identify factors associated with recent infection. A total of 2433, 2678, 2591, 2610 and 2541 participants were enrolled in 2011, 2012, 2013, 2014 and 2015, respectively. HIV incidence increased from 5.10% in 2011 to 6.62% in 2015 (*p* = 0.025). MSM who had an education level of junior high school or less (aOR = 1.472, *p* = 0.018), engaged in condomless anal sex in the past 6 months (aOR = 2.389, *p* < 0.001), did not have an HIV test in the past 12 months (aOR = 3.215, *p* < 0.001), and were currently infected with syphilis (aOR = 2.025, *p* = 0.001) were likely to be recently infected with HIV. HIV incidence is increasing among MSM in Jiangsu Province, China. Condom usage and HIV testing promotion should be prioritized when attempting to reduce HIV transmission among MSM in China.

## 1. Introduction

The HIV epidemic in China is now concentrated among men who have sex with men (MSM). Currently, a third of the HIV infections in China are accounted for from the MSM community [1]. A nationwide cross-sectional study conducted in 61 cities in China from February 2008 to September 2009 found that 4.9% of MSM were infected with HIV [2]. Data from national sentinel surveillance sites also showed that HIV prevalence among MSM has been increasing steadily, from 5.7% in 2010 to 7.5% in 2013 [3]. In several cities, HIV prevalence is higher than 15% among MSM (e.g., Chongqing [4] and Chengdu [5], all located in southwest China). However, HIV prevalence among other high-risk populations is below 1% (e.g., female sex workers and male STD (sexually transmitted disease) clinic attendees) or showing a decline (e.g., injecting drug users) [1].

Jiangsu Province, an economically developed region with more than 78 million people, is located in the southeastern part of China. Towards the end of September 2015, a total of 18,809 HIV positive cases had been diagnosed and reported [6]. Since the first cluster of HIV cases were detected among MSM in a bathhouse in 2006 [7], increasing numbers of HIV positive MSM cases have been detected throughout the province in the past decade. This statistic is similar to the national trend. HIV incidence among MSM was 3.36 per 100 person-years in an open cohort study conducted from 2008 to 2010 in Nanjing (the capital city of Jiangsu Province) [8]. In two other major cities, baseline HIV prevalence was 16.0% and 13.6%, respectively, with observed HIV incidence of 13.59 and 12.62 per 100 person-years [9]. However, HIV incidence throughout the province is still unknown.

Estimating HIV incidence is important to understand the current status of transmission dynamics, identify high-risk populations, monitor prevention efforts, and target resources at programs that are most effective in reducing transmissions [10]. However, it is difficult to accurately and widely estimate HIV incidence using the prospective cohort study method or the mathematical modelling method. The prospective cohort study method is time-consuming, expensive, and potentially biased. The applicable scope of the pure mathematical modelling method is limited. The inaccurate prevalence and mortality measurements may lead to deviation between estimated and actual incidence of HIV [10]. In 2002, Parekh et al. established a new serological testing algorithm for recent HIV seroconversion, using a branched peptide that included gp41 immunodominant sequences from HIV-1 subtypes B, E and D, named the BED method (IgG-capture BED-EIA) [11]. The BED method can indirectly measure increased quantity (proportion) of HIV-IgG in the serum and identify whether a person was infected with HIV within approximately six months and estimate the incidence of HIV. The BED method has shown that most HIV subtypes have similar windows of infection [10]. There were several studies based on this method all over the world [12,13]. Herein, we used the BED method to find recent HIV infections from five consecutive yearly surveys, examine the trends in HIV incidence, and identify factors associated with recent HIV infection among MSM in Jiangsu Province, China.

## 2. Materials and Methods

### 2.1. Study Design and Participants

A cross-sectional sentinel surveillance survey was conducted among MSM in Jiangsu Province annually from April to July from 2011 to 2015. MSM were enrolled in one of eight cities that acted as a survey site for the study. Eligible participants were biologically male, 18 years of age or older, and reported anal or oral sex with another male in the previous year. Each participant was recruited to have a questionnaire interview and blood specimen collection.

### 2.2. Sampling and Recruitment

Two convenience sampling methods were used to recruit MSM at survey sites: (1) At MSM venues. Staff from local Centers for Disease Control and Prevention (CDC) went to the MSM gathering venues to conduct on-site surveys. Owners of the venues (e.g., bars, discos, clubs and bathhouses) and MSM volunteers who had knowledge of sex-on-premise venues (e.g., parks or public bathrooms) referred interested and eligible participants to the recruiters or interviewers. Questionnaires were given and blood samples were taken at these venues; (2) Internet-based sampling. Participants were recruited by local CDC staff in partnership with local community-based organizations (CBOs) via internet-based recruitment. Information including the participants’ eligibility, counseling and testing sites, survey period, and contact number for inquiry was disseminated through the most well-known local gay websites and most popular QQ chat room groups (QQ is the most widely used chat room/messenger software in China) to invite MSM to participate. Eligible participants came to the local CDC to complete the questionnaires and blood draws.

All participants provided signed consent forms. Participants’ cell phone numbers or QQ numbers (email addresses) were obtained for HIV result notifications and referrals to HIV-related resources and services. In order to prevent duplicate testing, the same interviewers were staffed at each study site to identify participants (face, special characteristics), ask them if they received this survey in the past twelve months, and input participants’ cell phone number and/or QQ number to check duplication before conducting interviews. For participants found trying to test again, research staff referred them to the HIV testing clinic of their local CDC for HIV testing and counseling.

Participants were provided a reimbursement of 50 RMB (≈8 USD) for their transportation and meal costs. They were also informed of local HIV-related resources and services, which they could access in the future if needed.

### 2.3. Measures

Participants were interviewed face-to-face by interviewers who completed survey training each year. The questionnaire included socio-demographic information (e.g., age, marital status, registered residence, education level), recruitment source of participants, HIV knowledge questions, and HIV related behaviors (condomless anal sex with male partners in the past 6 months, HIV testing in the past 12 months, and history of sexually transmitted infections (STIs) in the past 12 months). In the study, eight questions were used to assess HIV-related knowledge of the participants, where comprehensive knowledge was defined as correctly answering six or more questions. Condomless anal sex was defined as failure to consistently use condoms with male partners in the past 6 months [14].

A volume of 3–5 mL venous blood was collected from each participant for HIV and syphilis testing. Plasma HIV antibodies were tested by an enzyme-linked immunosorbent assay (ELISA, Zhuhai Livzon Diagnostics Inc., Zhuhai, China). For those with HIV positive results, the same blood samples were retested with another ELISA reagent (Beijing Wantai Biological Pharmacy Enterprise Co., Ltd., Beijing, China). If both tests were positive, participants were contacted by CDC staff to draw a new blood sample again for confirmatory testing (WB, western blot assay, MP Biomedical Asia Pacific Pte. Ltd., Singapore. If lost to follow-up, the first specimen was used for confirmatory testing.). BED assay (Sedia BED HIV-1 incidence EIA, Sedia Biosciences Corporation, Portland, OR, USA) was conducted for all HIV antibody confirmatory positive cases except for unambiguous previously diagnosed cases (e.g., AIDS cases, diagnosed cases more than 6 months ago and patients receiving antiretroviral therapy). Syphilis antibody was tested by an ELISA reagent (Beijing Wantai, Beijing, China). The toluidine red untreated serum test (TRUST, Beijing Wantai) was used for confirmation of syphilis on ELISA positive samples. Syphilis infection was defined as having two positive tests.

BED assay was performed according to instructions provided by manufactory. The optical density (OD) of test specimens were normalized by a ratio using a calibrator (ODn, specimen OD/calibrator OD) to minimize internal variations. Those samples with ODn of 1.2 or less were tested again in triplicate and the median values were used for evaluation. Samples with ODn of 0.8 or less were considered to be from individuals with recent infection, with seroconversion having occurred within the previous 168 days (95% CI, 155–184 days) [13]. The BED-estimated HIV incidences were calculated by a Microsoft Excel-based program that was prepared for the study according to Sedia’s instructions.

HIV incidence is estimated by:

(1) $$I=\frac{F\times \left(365/w\right)\times R}{N\prime +F\times \left(365/w\right)\times R/2}\times 100\%$$

F is adjustment factor which is calculated by:

(2) $$F=\frac{\left(R/P\prime \right)+\gamma -1}{\left(R/P\prime \right)\times \left(\alpha -\beta +2\gamma -1\right)\alpha }\times 100\%$$

Here α is 0.8098, β is 0.7571, γ is 0.9315 and w is 168 days. R is the number of recent HIV infections; N is the number of HIV negative cases. N′ is the number of HIV negative cases after adjustment, N′ = N × (P′/P), P is the number of requested BED tests, P′ is the number of actual BED tests performed [15].

### 2.4. Statistical Analysis

Questionnaires at each survey site were double-entered and checked for accuracy using Epi data software (version 3.1, Epi Data Association, Odense, Denmark). Chi square tests and chi square trend tests were used to compare differences between years and observe trend over time. In the result tables of the chi square tests, linear-by-linear associations were the results of chi square trend tests. Factors associated with recent HIV infection were first assessed using univariate logistic regression analysis. Variables with *p* values less than 0.20 were entered into multivariable logistic regression models. Multivariable logistic regression analysis was conducted using forward method in order to determine the adjusted odds ratios (aORs). *p* values less than 0.05 were considered as a statistically significant difference. All analyses were conducted using SPSS software (version 19.0, SPSS Inc., Chicago, IL, USA).

### 2.5. Ethical Approval

The national HIV sentinel surveillance program and data analysis obtained ethical approval from the Institutional Review Board of China CDC (approval number X140121318).

## 3. Results

### 3.1. Trends in Socio-Demographic Characteristics and HIV Related Behaviors

Overall, 12,853 eligible MSM (2433, 2678, 2591, 2610 and 2541 in 2011, 2012, 2013, 2014 and 2015, respectively) were enrolled into the study. In this five-year period, MSM who were 25 to 39 year of age (*p* < 0.001), had an education level of college or higher (*p* < 0.001), were not Jiangsu registered residents (*p* < 0.001), or were recruited via the internet (*p* < 0.001) were more likely to participate in the surveys. Rates of comprehensive HIV knowledge increased from 92.5% in 2011 to 95.8% in 2015 (*p* = 0.015). Condomless anal sex with male partners in the past 6 months decreased from 44.6% in 2011 to 39.1% in 2015 (*p* < 0.001). Rates of HIV testing in the past 12 months showed a significant increase, from 46.4% in 2012 to 75.6% in 2015 (*p* < 0.001) (Table 1).

### 3.2. Trends in HIV and Syphilis Prevalence and HIV Incidence

A rise of HIV incidence was shown (*p* = 0.025), with 5.10% (95% CI: 3.77%–6.44%) in 2011, 5.85% (95% CI: 4.54%–7.17%) in 2012, 5.26% (95% CI: 4.01%–6.51%) in 2013, 7.76% (95% CI: 6.21%–9.31%) in 2014, and 6.62% (95% CI: 5.19%–8.06%) in 2015, respectively (Table 2). A total of 198 (8.1%), 256 (9.6%), 227 (8.8%), 277 (10.6%) and 256 (10.1%) participants tested HIV positive in the five years from 2011 to 2015, respectively, which explains the significant increase in HIV prevalence (*p* = 0.008). The prevalence of current syphilis infection ranges from 6.5% to 10.2%, but there was no consistent increase or decrease in the data over the years (*p* = 0.071) (Table 1).

### 3.3. Factors Associated with Recent HIV Infection

The potential factors associated with recent HIV infection were analyzed with HIV negative MSM in 2015 as control (MSM recruited were partly repetitive over the years). In the univariate analysis, there were significant differences in education level, comprehensive HIV knowledge, condomless anal sex in the past 6 months, HIV testing in the past 12 months, and current syphilis infection between MSM recently infected with HIV and those who were HIV negative. In the multivariate analysis, MSM who had an education level of junior high school or less (aOR = 1.472, 95% CI: 1.069–2.027, *p* = 0.018), had condomless anal sex in the past 6 months (aOR = 2.389, 95% CI: 1.835–3.110, *p* < 0.001), did not have an HIV test in the past 12 months (aOR = 3.215, 95% CI: 2.485–4.160, *p* < 0.001), and tested positive for syphilis (aOR = 2.025, 95% CI: 1.353–3.032, *p* = 0.001) were likely to be recently infected with HIV (Table 3).

## 4. Discussion

Though HIV incidence studies have a pivotal role in the HIV surveillance, these multi-year data sets are still limited in China. Our study is among the first to examine trends in HIV incidence over a period of five consecutive years among MSM in Jiangsu Province, China. Our observed incidence of HIV was more than 5% each year. It is slightly higher than that in Nanjing from two prospective cohort studies [8,16], lower than that in Yangzhou and Changzhou (all located in Jiangsu Province) from a prospective cohort study [9]. HIV incidence of Jiangsu Province are also comparable to other provinces with similar population metrics in China, e.g., Beijing (5.47, 12.37 and 6.86 per 100 person-years respectively, in the 6-month 12-month and 18-month follow-up visits) [17] and Zhejiang Province (3.52%, 6.63% and 6.33% respectively, in 2010, 2011 and 2012), the latter of which was also based their incidence levels on the BED method [18]. More importantly, in our study, a significant increase was observed over the five years. Rising incidence levels leads to people believing that HIV spread among MSM is becoming more serious, so they are contemplating creating more powerful control efforts and preventions.

While our study showed an increase in HIV incidence, it showed a decrease in the rates of engaging in condomless anal sex. This contrast is consistent with findings from other studies [4,19,20]. Possible explanations include that data of condom use were self-reported. The rate of condom usage was still very low. It is clear that promoting condom use alone is insufficient to curb the HIV epidemic among Chinese MSM. Other innovative and effective HIV prevention strategies should be developed and implemented. Recent initiatives have focused attention on HIV prevention measures including expanding HIV testing uptake in key populations, detecting HIV cases early, and linking HIV-infected individuals to care. More timely prescription of ART (antiretroviral therapy) is also important to prevent onward transmission of and decrease incidence of HIV at the population level [21,22,23]. However, in our study, MSM who engaged in condomless anal sex were more likely to be infected with HIV, which still emphasized the importance of promoting condom use.

Consistent with findings from other studies [2,24,25], MSM who had a low education level and had not been recently tested for HIV were more likely to be infected with HIV. Education level can affect individual’s occupation, income, and social status, which then influences one’s behavior. In addition, low education levels may mean one lacks AIDS/HIV knowledge. Correspondingly, MSM with less education were more likely to practice high-risk behaviors and be infected with HIV. Therefore, HIV testing intervention programs should pay increased attention to these subgroups of MSM and promote annual or more frequent testing for these high-risk groups. Previous studies also showed that social discrimination, fear of testing HIV positive, and lack of testing resources are major barriers to accessing HIV testing [26,27]. Intervention programs, such as health education campaigns and improved training of HIV testing counselors, should be used to create a friendly and supportive environment for MSM to take HIV tests confidently.

The literature has previously shown that syphilis increases the risk of HIV infection [16,25,28], which is consistent with our results. Basic research also shows that the opportunity for HIV to enter the body through different mechanisms is increased after syphilis infection [29]. The correlation of these two diseases shows that the screening and treating on STDs are very important to control for HIV among MSM. The preventions of STDs and HIV should be integrated with each other when constructing policy.

Different from most studies, we selected recently infected individuals for the case group for the study of associated factors to reduce the bias of changing behaviors of long-term infected patients. However, several limitations of this study should be noted. First, participants might provide socially desirable answers to sensitive questions due to the face-to-face interview survey mode employed. To minimize this bias, interviewers from all survey sites were trained annually and followed strict interviewing protocols. Second, overestimation of HIV incidence might exist due to misclassifications among long-term infections [10,30]. Removing unambiguous previously diagnosed cases before BED testing and adding an adjustment factor when estimating incidence are necessary. In addition, compared with the value of incidence, we should pay more attention to the trend. A new and similar method (limiting antigen avidity EIA) has been established and is being validated [31]. Finally, our results may not be generalizable to all MSM in the province and other regions.

## 5. Conclusions

In conclusion, this study demonstrates an increase in HIV incidence among MSM in Jiangsu province, during 2011–2015. Condom usage and HIV testing promotion should continuously take the top priority on the HIV prevention agenda for MSM in China, and the preventions of STDs and HIV should be integrated with each other when constructing new policy to reduce HIV transmission.

## Figures and Tables

**Table 1:** Trends in socio-demographic characteristics, HIV related behaviors and the prevalence of HIV and syphilis of MSM in sentinel surveillance surveys in Jiangsu province, China, 2011–2015.

Variable	2011	2012	2013	2014	2015	Trend Test *p*-Value
	(*N* = 2433)	(*N* = 2678)	(*N* = 2591)	(*N* = 2610)	(*N* = 2541)	
	*N* (%)	*N* (%)	*N* (%)	*N* (%)	*N* (%)	
**Age group (years)**						<0.001
18–24	887 (36.5)	858 (32.0)	918 (35.4)	926 (35.5)	721 (28.4)	
25–39	1136 (46.7)	1275 (47.6)	1233 (47.6)	1111 (42.6)	1299 (51.1)	
≥40	410 (16.9)	545 (20.4)	440 (17.0)	573 (22.0)	521 (20.5)	
Marital status *						0.176
Single, divorced or widowed	1637 (67.3)	1706 (63.8)	1846 (71.2)	1765 (67.6)	1709 (67.3)	
Married or cohabiting	796 (32.7)	966 (36.2)	745 (28.8)	845 (32.4)	831 (32.7)	
Education level *						<0.001
Junior high school or lower	542 (22.3)	622 (23.3)	534 (20.6)	527 (20.2)	463 (18.2)	
Senior high school	987 (40.6)	969 (36.3)	1019 (39.3)	956 (36.6)	840 (33.1)	
College or higher	904 (37.2)	1078 (40.4)	1038 (40.1)	1127 (43.2)	1238 (48.7)	
Registered residence *						<0.001
Jiangsu province	1864 (76.6)	2127 (79.6)	1953 (75.4)	1907 (73.1)	1907 (75.1)	
Other province	568 (23.4)	545 (20.4)	638 (24.6)	703 (26.9)	633 (24.9)	
Recruitment source of participants *						<0.001
venues	1460 (60.1)	1803 (67.5)	1363 (52.6)	1312 (50.3)	1235 (48.7)	
Internet	970 (39.9)	869 (32.5)	1227 (47.4)	1297 (49.7)	1303 (51.3)	
**Comprehensive HIV knowledge**	2250 (92.5)	2532 (94.5)	2410 (93.0)	2395 (91.8)	2435 (95.8)	0.015
Condomless anal sex, past 6 months *	1078 (44.6)	1075 (40.4)	1060 (41.0)	1075 (41.3)	993 (39.1)	0.001
STI, past 12 months (self-reported) *	161 (6.7)	119 (4.5)	93 (3.6)	153 (5.9)	159 (6.3)	0.555
HIV testing, past 12 months *	1458 (59.9)	1242 (46.4)	1364 (52.6)	1371 (52.5)	1854 (75.6)	0.001
**Current syphilis infection**	249 (10.2)	196 (7.3)	169 (6.5)	204 (7.8)	212 (8.3)	0.071
**HIV positive**	198 (8.1)	256 (9.6)	227 (8.8)	277 (10.6)	256 (10.1)	0.008

Notes: * Data missing or refusal; STI, sexually transmitted infections.

**Table 2:** Trends in HIV incidence among MSM in sentinel surveillance surveys in Jiangsu province, China, 2011–2015.

Year	Negative (*N*)	Adjusted Negative (*N′*)	Requested BED Testing (*P*)	Actual BED Testing (*P′*)	Recent (*R*)	Incidence (%) (95% CI) (*I*)
2011	2228	2047	172	158	56	5.10 (3.77–6.44)
2012	2416	2384	228	225	76	5.85 (4.54–7.17)
2013	2356	2356	210	210	68	5.26 (4.01–6.51)
2014	2329	2311	256	254	96	7.76 (6.21–9.31)
2015	2280	2271	241	240	82	6.62 (5.19–8.06)

Notes: The numbers of WB indeterminate samples were 7, 6, 8, 4, 5, respectively, in 2011, 2012, 2013, 2014, 2015; The numbers of unambiguous previously diagnosed cases were 26, 28, 17, 21, 15, respectively; The reasons of not testing BED (difference between P and P′) were samples missing or poor quality.

**Table 3:** Univariate and multivariate analysis of factors associated with recent HIV infection among MSM in sentinel surveillance surveys in Jiangsu province, China, 2011–2015.

Variable	Negative *N* (%)	Recent *N* (%)	OR (95% CI)	*p*-Value	aOR (95% CI)	*p*-Value
**Age group (years)**						
18–24	652 (28.6)	133 (35.2)	1.243 (0.913–1.693)	0.167		
25–39	1177 (51.6)	171 (45.2)	0.885 (0.660–1.188)	0.417		
≥40	451 (19.8)	74 (19.6)	1			
**Marital status**						
Single, divorced or widowed	1529 (67.1)	263 (69.6)	1.122 (0.886–1.420)	0.340		
Married or cohabiting	750 (32.9)	115 (30.4)	1			
**Education level**						
Junior high school or less	394 (17.3)	100 (26.5)	1.799 (1.366–2.370)	<0.001	1.472 (1.069–2.027)	0.018
Senior high school	766 (33.6)	120 (31.7)	1.110 (0.861–1.432)	0.420	0.990 (0.738–1.328)	0.944
College or higher	1120 (49.1)	158 (41.8)	1		1	
**Registered residence**						
Jiangsu province	1720 (75.5)	270 (71.4)	1			
Other province	559 (24.5)	108 (28.6)	1.231 (0.966–1.569)	0.094		
**Recruitment source of participants**						
Venues	1133 (49.8)	177 (46.9)	1			
Internet	1144 (50.2)	200 (53.1)	1.119 (0.900–1.392)	0.312		
**Comprehensive HIV knowledge**						
No	92 (4.0)	29 (7.7)	1.976 (1.282–3.045)	0.002		
Yes	2188 (96.0)	349 (92.3)	1			
**Condomless anal sex, past 6 months**						
No	926 (52.1)	101 (31.7)	1			
Yes	852 (47.9)	218 (68.3)	2.346 (1.820–3.023)	<0.001	2.389 (1.835–3.110)	<0.001
**STI, past 12 months (self-reported)**						
No	2146 (94.3)	349 (92.6)	1			
Yes	130 (5.7)	28 (7.4)	1.324 (0.867–2.023)	0.194		
**HIV testing, past 12 months**						
No	495 (22.5)	178 (47.8)	3.162 (2.521–3.967)	<0.001	3.215 (2.485–4.160)	<0.001
Yes	1706 (77.5)	194 (52.2)	1		1	
**Current syphilis infection**						
No	2117 (92.9)	324 (85.7)	1			
Yes	163 (7.1)	54 (14.3)	2.165 (1.557–3.009)	<0.001	2.025 (1.353–3.032)	0.001
